# cAMP-MFN2 signaling suppresses cochlear cell senescence and age-related hearing loss

**DOI:** 10.3389/fimmu.2025.1715738

**Published:** 2025-11-26

**Authors:** Yu Liu, Jinyuan Cao, Jie Cui, Haitong Chen, Shujing Qin, Qi Li, Zhongwu Su

**Affiliations:** 1 The First School of Clinical Medicine, Southern Medical University, Guangzhou, China; 2 Department of Otolaryngology, Nanfang Hospital, Southern Medical University, Guangzhou, China

**Keywords:** age-related hearing loss (ARHL), cAMP, mitofusin2, mitochondrial dynamics, inflammation

## Abstract

**Introduction:**

Age-related hearing loss (presbycusis) is the most common sensory deficit in the elderly, yet it lacks effective pharmacological treatments. The decline of the cyclic AMP (cAMP) signaling pathway is implicated in aging, but its functional role in the auditory system remains largely unknown.

**Methods:**

To investigate whether enhancing cAMP signaling could counteract presbycusis and elucidate the underlying molecular mechanism, we used a D-galactose-induced cellular senescence model and an in vivo aged mouse model. The effects of the cAMP analog dibutyryl-cAMP (dbcAMP) on cellular senescence, inflammation, mitochondrial function, and cochlear structure were evaluated. The role of Mitofusin-2 (MFN2) was further assessed using siRNA-mediated knockdown.

**Results:**

dbcAMP potently suppressed cellular senescence and the associated inflammatory phenotype. In addition, cAMP treatment alleviated mitochondrial dysfunction, as indicated by improved mitochondrial bioenergetics and morphology. The anti-senescence effects of cAMP were significantly blunted upon siRNA-mediated knockdown of Mfn2, establishing MFN2 as a key, though perhaps not the sole, component of this protective pathway. Importantly, systemic administration of dbcAMP to aged mice significantly preserved hearing function and protected cochlear hair cells. This in vivo protection was accompanied by an upregulation of cochlear MFN2 and a coordinated suppression of senescence and inflammation markers.

**Discussion:**

Our study reveals a critical protective role for the cAMP-MFN2 axis in auditory aging by suppressing cellular senescence. These findings identify this axis as a novel and promising therapeutic target for the treatment of age-related hearing loss.

## 1 Introduction

Hearing is a fundamental medium through which we perceive the world, connect with others, and construct memory and identity. The gradual loss of this faculty with age is not only a clinical concern but also a profound human vulnerability. Clinically known as presbycusis, this condition represents the most common sensory impairment in the aging population, contributing significantly to social isolation and an increased risk of dementia (1, 2). While the progressive loss of cochlear hair cells is the central pathological feature (3), the molecular mechanisms driving this degeneration remain largely unelucidated.

The cyclic AMP (cAMP) signaling pathway is a ubiquitous and evolutionarily conserved system that regulates a wide range of cellular processes. Modulation of the cAMP signaling pathway has been demonstrated to directly influence the progression of aging across various tissues (4–7). Moreover, its dysregulation has been linked to various forms of hearing impairment (8, 9). Previous cochlear metabolomic studies have shown that noise-induced hearing loss is associated with significant alterations in the cAMP signaling pathway (10). In our previous studies, we also observed dysregulation of the cAMP pathway in the aged cochlea (11). However, the downstream cellular mechanisms that link the attenuation of cAMP signaling to the auditory aging phenotype remain to be elucidated.

Two of the fundamental hallmarks of cellular aging are mitochondrial dysfunction and a state of chronic inflammation (12, 13). The integrity of the mitochondrial network, which is dynamically regulated by fusion and fission events, is essential for cellular homeostasis. Its fragmentation is a common feature of senescent cells, actively fueling the inflammaging process (14, 15). The cAMP pathway is increasingly recognized as a key regulator of both these processes. It is known to modulate various aspects of mitochondrial function and can exert anti-inflammatory effects in several contexts (16–19). While its precise influence on the key proteins governing mitochondrial dynamics remains incompletely understood, a regulatory link between the transcription factor CREB, a primary downstream target of cAMP, and MFN2 has been proposed in other disease models, where it modulates MFN2-mediated mitophagy (20). Given that cAMP signaling can influence MFN2-dependent mitochondrial quality, it is plausible that it also regulates the broader mitochondrial dynamics of fusion and fission. Considering the relationship between mitochondrial dynamics and aging, a comprehensive understanding of the physiological processes influenced by the cAMP-MFN2 axis in the aging pathological environment of the cochlea remains a critical unanswered question.

In this study, we sought to bridge this knowledge gap. We hypothesized that enhancing cAMP signaling could counteract age-related hearing loss by attenuating cellular senescence in an MFN2-dependent manner. Our findings reveal that cAMP upregulates MFN2, thereby suppressing the senescence phenotype and preserving cochlear function in aged mice. This work identifies the cAMP-MFN2 axis as a novel therapeutic target for presbycusis, providing a promising new strategy for addressing this challenging condition.

## 2 Materials and methods

### 2.1 Cell culture and treatments

The House Ear Institute-Organ of Corti 1 (HEI-OC1, kindly provided by Prof. Hongzheng Zhang (Zhujiang Hospital, Guangzhou, China) cell line was cultured in high-glucose Dulbecco’s Modified Eagle Medium (DMEM; Gibco, C11995500BT) supplemented with 10% fetal bovine serum (FBS; Excell, FSP500) and 1% penicillin-streptomycin (Beyotim, C0222), at 33 °C in a humidified atmosphere of 10% CO_2_.

Cellular senescence was induced by treatment with D-galactose (D-gal; Sigma-Aldrich) at a final concentration of 40mg/ml for 48 hours. This concentration of D-gal is commonly used to induce cellular senescence, as reported in previous studies (21). To assess the effects of cAMP, cells were co-treated with D-gal and dbcAMP (MedChemExpress) at 1 mM for the indicated times. dbcAMP (dibutyryl-cAMP), a membrane-permeable and hydrolysis-resistant analog of cAMP, was used instead of native cAMP, which is rapidly degraded by phosphodiesterases and has poor membrane permeability (22, 23). The 1 mM concentration of dbcAMP was chosen based on previous studies that demonstrated its effectiveness and non-toxicity (24).

### 2.2 siRNA transfection

Small interfering RNAs (siRNAs) targeting mouse *Mfn2* (si*Mfn2*) and a non-targeting negative control siRNA (siNC) were synthesized by GenePharma. The sequences: sense(5’-3’): CUGCCAUGAACAAGAAAGUTT; antisense(5’-3’): ACUUUCUUGUUCAUGGCAGTT. HEI-OC1 cells were transfected with 50 nM of siRNA using siRNA-mate plus (GenePharma) according to the manufacturer’s protocol. At 48 hours post-transfection, cells were harvested for subsequent assays.

### 2.3 RNA extraction and quantitative real-time polymerase chain reaction

Total RNA was extracted from cells or cochlear tissues using a Total RNA Rapid Extraction Kit (FINDROP, RZ319-04). Complementary DNA (cDNA) was synthesized using a high-capacity cDNA reverse transcription kit (Vazyne). RT-PCR analysis was performed by a StepOnePlus system (Vazyne) using the SYBR-Green Real-Time PCR Master Mix kit (Vazyne). Relative gene expression was calculated using the 2^−ΔΔCt method, with β-actin as the internal control. Primer sequences are provided in **Supplementary Table S1**.

### 2.4 Western blot analysis

Cells or cochlear tissues were lysed in RIPA buffer (Beyotime, P0013B) supplemented with protease and phosphatase inhibitors (NCM, P002). Protein concentration was determined using a BCA Protein Assay Kit (Beyotime, P0009). Equal amounts of protein were separated by SDS-PAGE and transferred to PVDF membranes (Merck Millipore, IPVH00010).

Membranes were blocked with 5% non-fat milk in TBST for 1 hour at room temperature and incubated overnight at 4 °C with primary antibodies against MFN2 (SANTA CRUZ, XX-1, 1:500), p21 (Abcam, AB188224, 1:1000), p-CREB (Abcam, AB32096, 1:1000), CREB (SANTA CRUZ, sc-271, 1:500), p-p65 (Abmart, TP56372, 1:1000), p65 (Abmart, T55034, 1:1000), IL-6 (SANTA CRUZ, XX-1, 1:500), TNF-α (Proteintech, 17590-1-AP, 1:1000), and β-actin (Beyotime, AA128-1,1:1000). After washing, membranes were incubated with HRP-conjugated secondary antibodies (Proteintech) for 1 hour at room temperature. Protein bands were visualized with an ECL kit (Merck Millipore) and imaged using an Amersham Imager 600, GE Life Sciences. Band intensities were quantified using ImageJ software.

### 2.5 Cellular staining and mitochondrial assays

Senescence-Associated β-Galactosidase (SA-β-gal) Staining: SA-β-gal activity was assessed using a commercial staining kit (Beyotime, C0602). Senescent cells were quantified from at least three random microscopic fields.

Mitochondrial Morphology and Membrane Potential: Cells were stained with MitoTracker Red CMXRos (Beyotime, 100 nM, 15min) for mitochondrial morphology and with JC-1 dye (MCE, 3520-43-2) for membrane potential. Images were obtained by a confocal microscopy (Nikon, Japan), and JC-1 red/green fluorescence ratios were quantified with ImageJ software.

### 2.6 ATP content analysis

Cellular ATP levels were determined using an Enhanced ATP Assay Kit (Beyotime, China) following the manufacturer’s instructions. Chemiluminescence was detected using a Synergy Neo2 Multi-Mode Microplate Reader (BioTek Instruments, USA). ATP concentrations were calculated based on a standard curve generated from serial ATP standards. Total protein content in each sample was quantified using a BCA Protein Assay Kit (Beyotime, China), and the ATP level was expressed as nmol per mg of protein. ATP values were further normalized to the control group and presented as relative ATP levels.

### 2.7 Animals and *in vivo* treatments

Male C57BL/6J mice were purchased from GemPharmatech and housed in a specific-pathogen-free (SPF) facility under a 12-hour light/dark cycle with ad libitum access to food and water. Young mice (5 weeks old) and aged mice (34–36 weeks old) were used for the experiments as indicated.

For the therapeutic study, aged mice were randomly assigned to vehicle or treatment groups (n = 10 per group at the start). The treatment group received intraperitoneal (i.p.) injections of dbcAMP (MCE, 16980-89-5) at a dose of 50 mg/kg body weight, dissolved in sterile saline (0.9% NaCl). The control group received i.p. injections of an equivalent volume of saline. Injections were administered every other day for 28 consecutive days. Mice were euthanized by cervical dislocation for cochlear tissue harvesting. This dose was selected based on previous studies demonstrating the effectiveness and safety of dbcAMP at 50 mg/kg via intraperitoneal injection (22).

Animals were randomly allocated to groups, and investigators performing ABR measurements and histological analyses were blinded to group assignments. All animal procedures were approved by the Institutional Animal Care and Use Committee of Southern Hospital, Southern Medical University, Guangzhou, China (Approval No. IACUC-LAC-20250728-001) and were performed in strict accordance with the National Institutes of Health Guide for the Care and Use of Laboratory Animals. All efforts were made to minimize animal suffering.

### 2.8 Auditory brainstem response

Auditory brainstem response (ABR) measurements were conducted to assess auditory function using a Tucker-Davis Technologies System 3 (TDT 3, Alachua, FL, USA). Mice were anesthetized with an i.p. injection of 1.25% avertin (AiBei, M2910). Body temperature was maintained at 37 °C using a heating pad. Briefly, mice were subcutaneously inserted with subdermal needle electrodes at the vertex of the skull (active), below the left ear (reference) and the right ear (ground) after being anesthetized. ABR tests were measured at 8, 16, 24, and 32 kHz. The average response to 512 stimuli was obtained through reducing the sound intensity at 5 dB intervals near the threshold, which was defined as the lowest stimulus level where a positive wave was evident.

### 2.9 Cochlear immunofluorescence

Cochleae were dissected and fixed in 4% paraformaldehyde (PFA) for 12 hours. After decalcification in 10% EDTA for three days, the cochlea was embedded in O.C.T., cryosectioned at a 10 μm thickness, and stored at -20°C. Samples were permeabilized with 0.3% Triton X-100 (Sigma-Aldrich), blocked with 10% goat serum (Solarbio), and incubated overnight at 4 °C with primary antibody against p-CREB (Abcam, AB32096, 1:200), CREB (SANTA CRUZ, sc-271, 1:50). After washing, tissues were incubated with the corresponding secondary antibody at room temperature for 1h. Images were acquired using a confocal microscope (Nikon, Japan).

### 2.10 Cochlear basilar membrane myosin VIIa staining

The basilar membrane was permeabilized with 3% Triton X-100 at room temperature for 20 min, and blocked with 10% goat serum (Solarbio, China, SL038) at room temperature for 1 h. The organs of corti were then incubated with myosin VIIa (1:200, Proteus Biosciences, USA, 25-6790) at 4 °C for 24 h. After washing with PBS, the tissues were incubated with the secondary antibody at room temperature for 1h. The samples were observed under a confocal microscope (Nikon, Japan).

### 2.11 Statistical analysis

All quantitative data are expressed as mean ± standard deviation (SD). Statistical analyses were performed using GraphPad Prism 9 (GraphPad Software, USA). Comparisons between two groups were made using unpaired two-tailed Student’s t-tests. For multiple groups, one-way ANOVA with Tukey’s *post hoc* test was applied. A p-value < 0.05 was considered statistically significant. ImageJ was used for densitometric and fluorescence quantification. Each experiment was independently repeated at least three times. The number of biological replicates is specified in the figure legends.

## 3 Results

### 3.1 Exogenous cAMP mitigates cellular senescence *in vitro*

**Figure 1:**
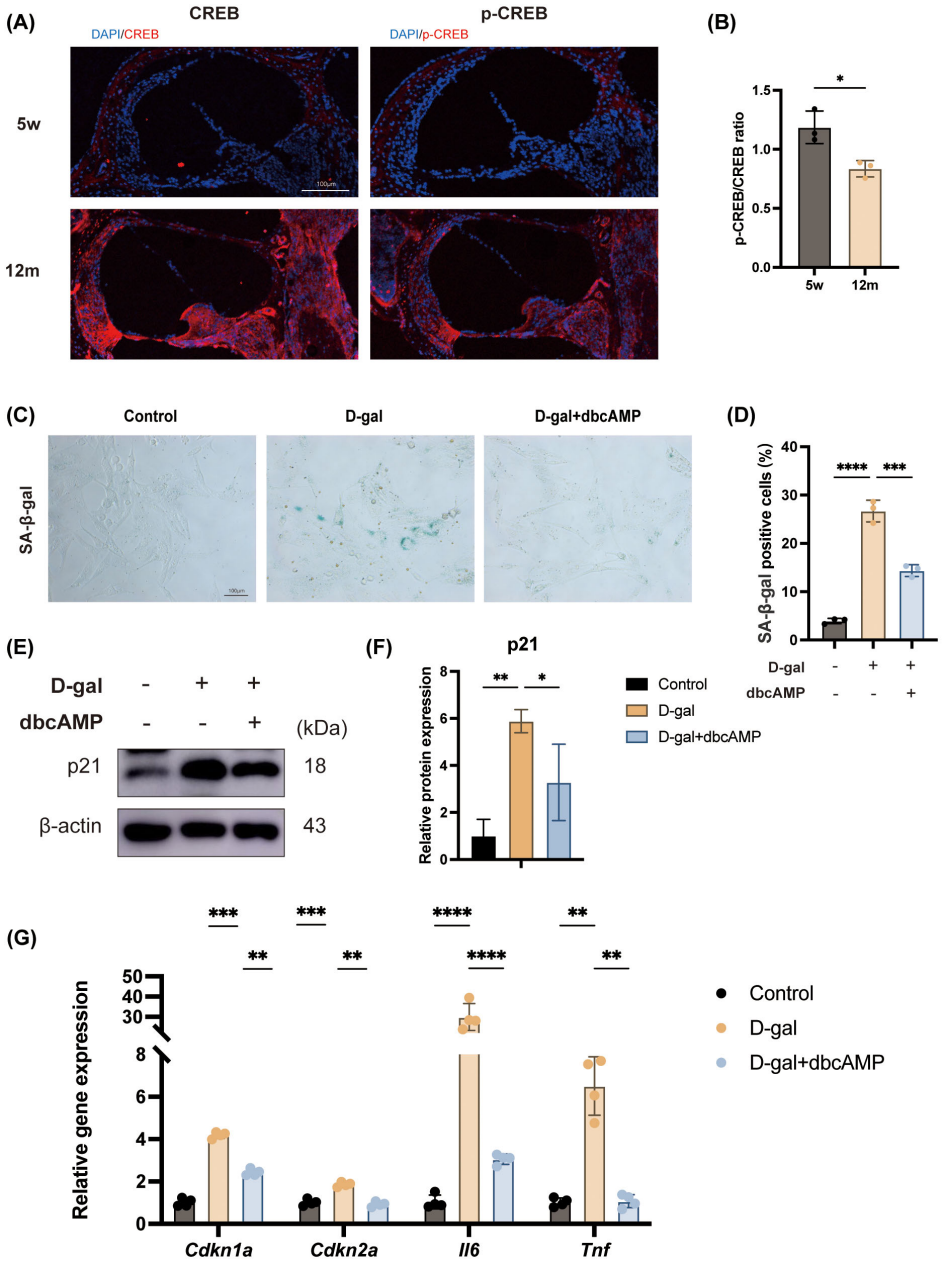
Exogenous cAMP mitigates cellular senescence *in vitro*. **(A, B)** Representative immunofluorescence staining of CREB and p-CREB in cochlear sections from young (5 weeks) and aged (12 months) mice, showing reduced p-CREB/CREB ratio with aging. Scale bar = 100μm; n = 3; student’s t-tests. **(C, D)** SA-β-gal staining of HEI-OC1 cells treated with D-gal in the presence or absence of dbcAMP for 48 hours. Scale bar = 100μm; n = 3. **(E, F)** Western blot analysis of p21 protein levels in control, D-gal-treated, and D-gal + dbcAMP-treated cells. β-actin served as loading control. n = 3. **(G)** qPCR analysis of senescence- and inflammation-related gene expression across groups, showing that dbcAMP reduces D-gal-induced upregulation of *Cdkn1a*, *Cdkn2a*, *Il6* and *Tnf*. n = 4. Unless otherwise specified, cells were treated with D-gal (40mg/ml) and/or dbcAMP (1 mM) for 48 hours. Data are presented as mean ± SD. *p < 0.05, **p < 0.01, ***p < 0.001, ****p < 0.0001, n.s. no statistical difference; one-way ANOVA.

To investigate the potential signaling pathways involved in age-related hearing loss, we first examined the activation status of the cAMP response element-binding protein (CREB), a key transcription factor downstream of cAMP signaling, in the murine cochlea. Representative immunofluorescence staining of cochlear sections from young (5 weeks) and aged (12 months) mice revealed a marked decrease in phosphorylated CREB (p-CREB) relative to total CREB in aged (12-month-old) mice compared to young (5-week-old) mice, suggesting an age-associated impairment of the cAMP signaling pathway in the cochlea (**Figures 1A, B**).

Based on this *in vivo* observation, we hypothesized that supplementing with exogenous cAMP could counteract senescence-related phenotypes. To test this, we established a D-galactose (D-gal)-induced senescence model. As assessed by senescence-associated β-galactosidase (SA-β-gal) staining, D-gal treatment led to a significant increase in the proportion of senescent cells compared to the control group. Notably, co-treatment with dibutyryl-cAMP (dbcAMP) effectively reversed this phenotype, markedly reducing the number of SA-β-gal-positive cells (**Figures 1C, D**).

To further substantiate the anti-senescence effect of cAMP at the molecular level, we measured the expression of key senescence and inflammation-related markers. Western blot analysis showed that the protein level of the cell cycle inhibitor p21 was substantially upregulated following D-gal exposure. This effect was significantly suppressed by the addition of dbcAMP (**Figures 1E, F**). Consistent with these findings, qPCR analysis revealed that D-gal induced a significant transcriptional upregulation of senescence-related genes (*Cdkn1a* and *Cdkn2a*) and pro-inflammatory cytokines (*Il6* and *Tnf*). Importantly, dbcAMP treatment significantly blunted the D-gal-induced expression of this entire panel of senescence- and inflammation-associated genes (**Figure 1G**).

Collectively, these results demonstrate that cochlear aging is correlated with diminished cAMP signaling activity and that exogenous cAMP can effectively mitigate the hallmarks of cellular senescence and the associated inflammatory phenotype caused by D-gal *in vitro*.

### 3.2 cAMP restores mitochondrial homeostasis and upregulates MFN2 expression in senescent cells

**Figure 2:**
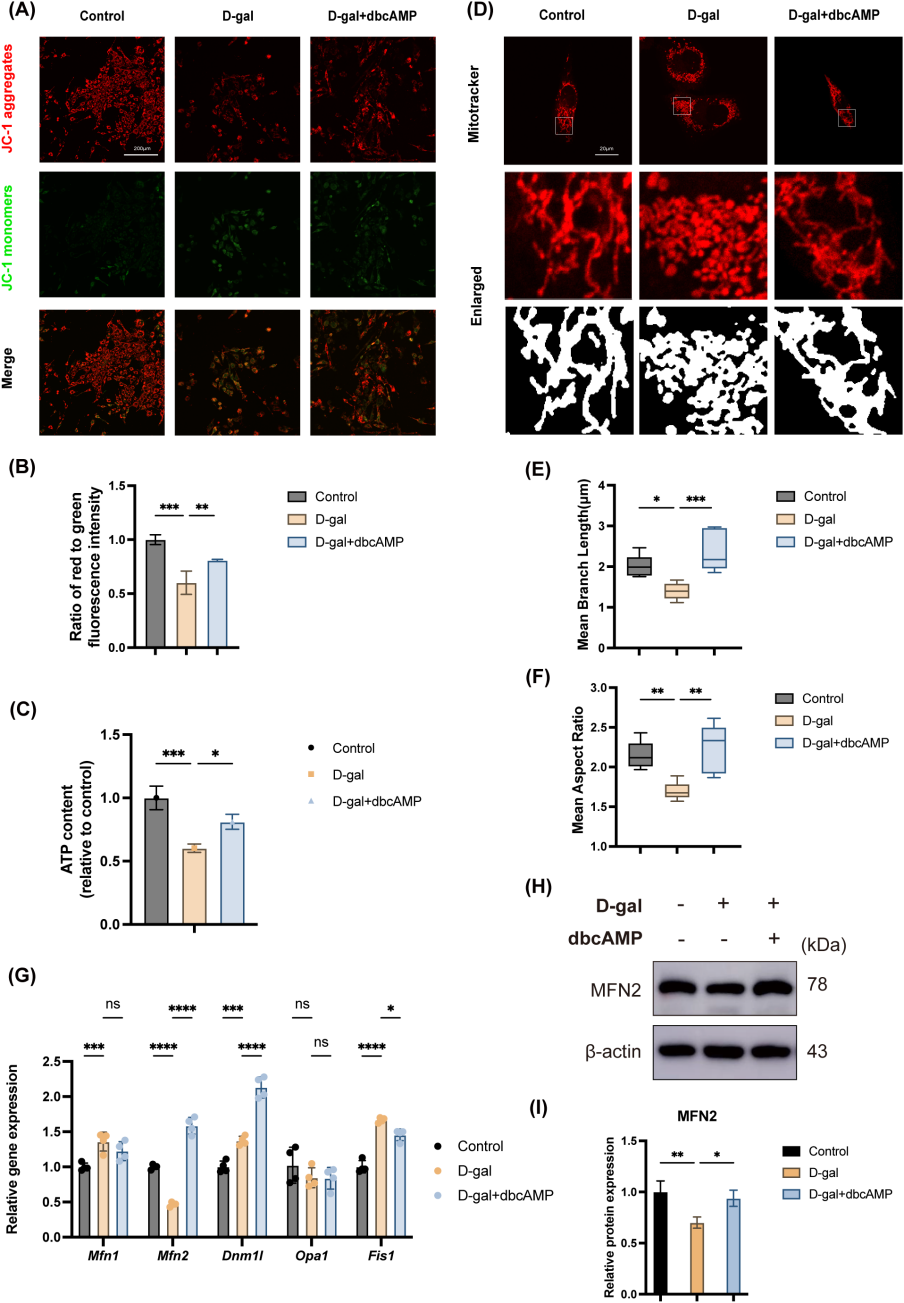
cAMP restores mitochondrial homeostasis and upregulates MFN2 expression in senescent cells. **(A, B)** Representative JC-1 staining showing mitochondrial membrane potential in control, D-gal-treated, and D-gal + dbcAMP-treated cells. D-gal treatment caused a significant decrease in the JC-1 red/green fluorescence intensity ratio, indicating a loss of mitochondrial membrane potential. Notably, this ratio was significantly increased following dbcAMP treatment, demonstrating a partial restoration of the membrane potential. Scale bar = 200μm; n = 3. **(C)** Quantification of cellular ATP levels in control, D-gal-treated, and D-gal + dbcAMP-treated cells. D-gal treatment significantly reduced ATP content compared with the control group, whereas dbcAMP treatment markedly restored ATP levels. n = 3. **(D–F)** Representative MitoTracker staining and enlarged images showing mitochondrial morphology. D-gal-treated cells exhibited a marked loss of the normal tubular mitochondrial network, displaying a predominantly fragmented and punctate mitochondrial morphology, whereas dbcAMP treatment markedly restored mitochondrial morphology. Scale bar = 20μm; n = 5. **(G)** qPCR analysis of mitochondrial dynamics-related genes (*Mfn1*, *Mfn2*, *Dnm1l*, *Opa1*, *Fis1*). D-gal reduced *Mfn2* expression, which was restored by dbcAMP. n = 4. **(H, I)** Western blot analysis of MFN2 protein in control, D-gal-treated, and D-gal + dbcAMP-treated cells, confirming that dbcAMP restores MFN2 levels decreased by D-gal treatment. β-actin served as a loading control. n = 3. Unless otherwise specified, cells were treated with D-gal (40mg/ml) and/or dbcAMP (1 mM) for 48 hours. Data are presented as mean ± SD. *p < 0.05, **p < 0.01, ***p < 0.001, ****p < 0.0001, n.s. no statistical difference; one-way ANOVA.

Given the central role of mitochondria in the aging process, we next sought to determine whether the anti-senescence effect of cAMP observed in **Figure 1** was associated with the preservation of mitochondrial integrity. To investigate the effect of dbcAMP on mitochondrial function, we first assessed the mitochondrial membrane potential (MMP) using JC-1 staining. In D-gal-treated cells, a distinct shift from red-fluorescent J-aggregates to green-fluorescent J-monomers was observed, indicating a significant collapse of the MMP. Notably, treatment with dbcAMP partially but significantly restored the MMP, as evidenced by an increase in the red/green fluorescence intensity ratio (**Figures 2A, B**). Given that MMP is crucial for ATP synthesis, we next examined cellular ATP levels. Consistent with the MMP dissipation, cellular ATP production was significantly impaired in the D-gal group compared to the control. Importantly, co-treatment with dbcAMP markedly reversed this energy deficit and partially restored cellular ATP levels (**Figure 2C**).

We then examined mitochondrial morphology using MitoTracker staining. Consistent with mitochondrial dysfunction, cells exposed to D-gal exhibited severe mitochondrial fragmentation, characterized by fragmented mitochondria with significantly shortened branch length and a reduced aspect ratio. In stark contrast, dbcAMP treatment markedly reversed these morphological abnormalities, promoting the formation of elongated and interconnected mitochondrial networks characteristic of healthy cells (**Figures 2D–F**).

Given the significant alterations in mitochondrial function and morphology after dbcAMP treatment, we next analyzed the expression of key genes involved in mitochondrial dynamics, including Mitofusin 1 (*Mfn1*), *Mfn2*, dynamin-1-like (*Dnm1l*), optic atrophy 1 (*Opa1*), and fission 1 *(Fis1*). These genes have been widely studied for their roles in regulating mitochondrial morphology. qPCR analysis revealed that among the tested genes, the expression of *Mfn2* was significantly downregulated by D-gal treatment. Importantly, this reduction was completely restored to control levels upon co-treatment with dbcAMP (**Figure 2G**). To confirm this finding at the protein level, we performed Western blot analysis. The results clearly demonstrated that MFN2 protein levels were diminished in D-gal-treated cells and robustly rescued by dbcAMP administration (**Figures 2H, I**).

Together, these findings indicate that the protective effects of cAMP against cellular senescence are tightly linked to the restoration of mitochondrial homeostasis, including both function and morphology, and are strongly correlated with the upregulation of the key mitochondrial fusion protein MFN2.

### 3.3 MFN2 is required for the anti-senescence and anti-inflammatory effects of cAMP

**Figure 3:**
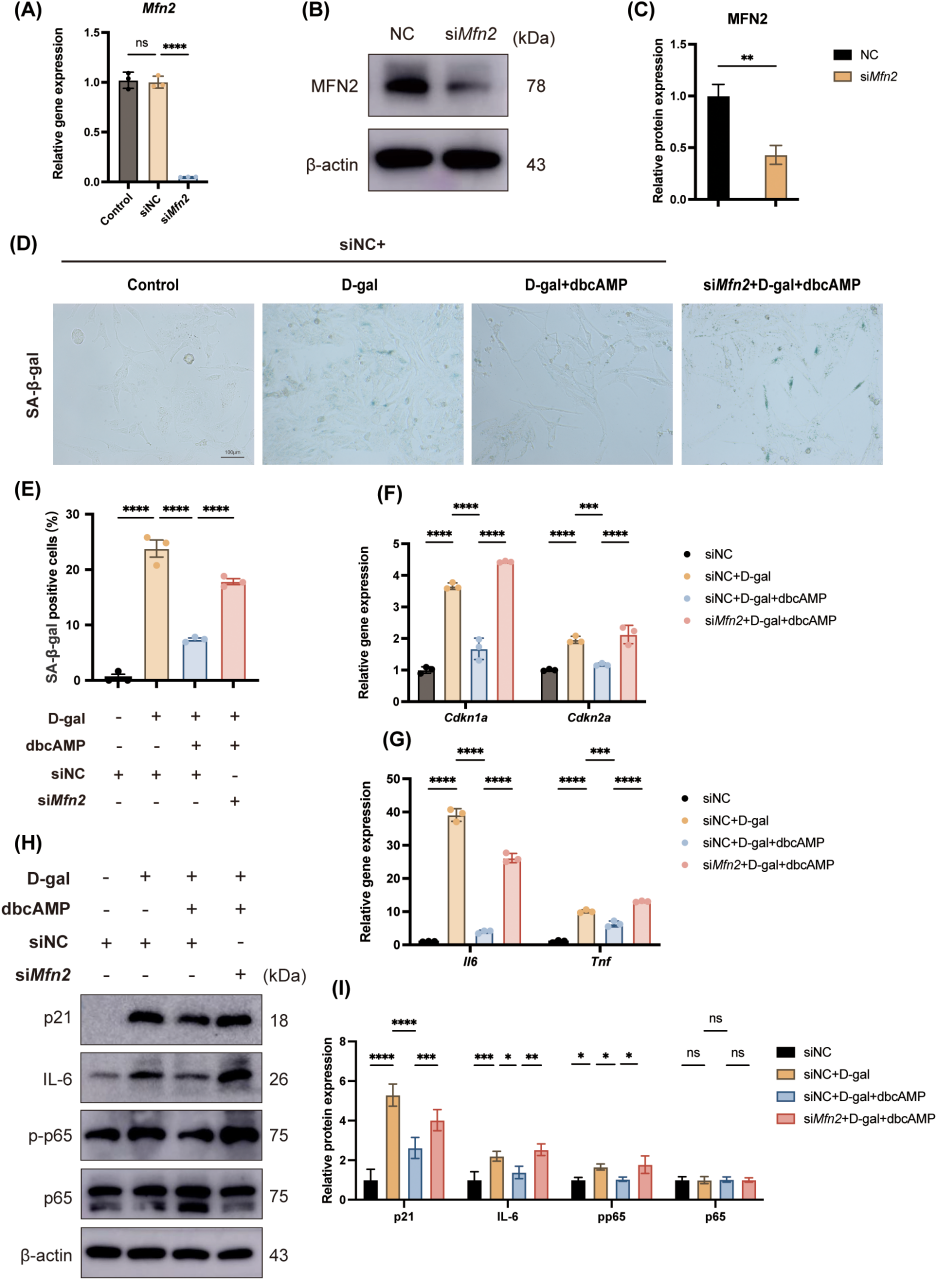
MFN2 is required for the anti-senescence and anti-inflammatory effects of cAMP. **(A)** qPCR analysis showing efficient knockdown of *Mfn2* mRNA by siRNA. n = 3. **(B, C)** Western blot validation of MFN2 knockdown. β-actin served as a loading control. n = 3; student’s t-tests. **(D, E)** SA-β-gal staining of siNC, siNC + D-gal-treated, siNC + D-gal + dbcAMP-treated, and si*Mfn2* + D-gal + dbcAMP-treated cells, showing increased senescent cell proportion upon MFN2 knockdown. Scale bar = 100μm; n = 3. **(F, G)** qPCR analysis of senescence-associated genes and inflammatory cytokines. D-gal induced significant upregulation of *Cdkn1a*, *Cdkn2a*, *Il6*, *Tnf*, which was suppressed by dbcAMP in siNC cells but restored upon *Mfn2* knockdown. n = 3. **(H, I)** Western blot analysis of p21, IL-6, p-p65, and p65 proteins. D-gal increased p21, IL-6, and p-p65 expression; dbcAMP treatment reduced their levels in siNC cells, while MFN2 protein knockdown abolished the protective effect of dbcAMP. n = 3. For all experiments in this figure, cells were first transfected with siNC or si*Mfn2* for 48 hours prior to subsequent treatments. Unless otherwise specified, cells were treated with D-gal (40mg/ml) and/or dbcAMP (1 mM) for 48 hours. Data are presented as mean ± SD. *p < 0.05, **p < 0.01, ***p < 0.001, ****p < 0.0001, n.s. no statistical difference; one-way ANOVA.

Our findings thus far strongly suggested that MFN2 is a critical downstream effector in the cAMP-mediated protective pathway. To definitively establish this causal link, we performed loss-of-function experiments using a specific siRNA targeting *Mfn2*. The efficacy of the siRNA was validated at both the mRNA and protein levels, demonstrating a significant and robust knockdown of *Mfn2* expression compared to the negative control (siNC) (**Figures 3A–C**).

We then investigated whether MFN2 was necessary for the anti-senescence effects of cAMP. As expected, dbcAMP treatment significantly reduced the proportion of SA-β-gal-positive cells in the D-gal-treated siNC group. Strikingly, this protective effect was completely abolished in cells where *Mfn2* was knocked down; the proportion of senescent cells in the si*Mfn2* + D-gal + dbcAMP group was increased significantly compared to the siNC + D-gal + dbcAMP-treated group (**Figures 3D, E**).

This observation was further corroborated by the expression analysis of senescence and inflammation-related genes. In siNC cells, dbcAMP effectively attenuated the D-gal-induced upregulation of *Cdkn1a* (p21), *Cdkn2a* (p16), *Il6*, and *Tnf*. However, in MFN2-deficient cells, dbcAMP lost its ability to suppress these genes, and their expression levels remained high (**Figures 3F, G**).

To further probe the underlying inflammatory signaling, we examined key protein markers. Western blot analysis revealed that dbcAMP treatment in siNC cells not only reduced the expression of p21 and IL-6 but also markedly inhibited the phosphorylation of p65 (p-p65), while the total p65 levels remained unchanged. Consequently, the p-p65/p65 ratio was significantly reduced by dbcAMP treatment, a key indicator of NF-κB pathway activation. Critically, the knockdown of MFN2 greatly abrogated the ability of dbcAMP to suppress the expression of p21, IL-6, and the activation of p65 (**Figures 3H, I**).

Taken together, these results provide compelling evidence that MFN2 is an indispensable mediator for the anti-senescence and anti-inflammatory effects of cAMP, and that this protection is associated with the inhibition of the NF-κB signaling pathway.

### 3.4 cAMP treatment rescues auditory function and suppresses cochlear senescence in aged mice

**Figure 4:**
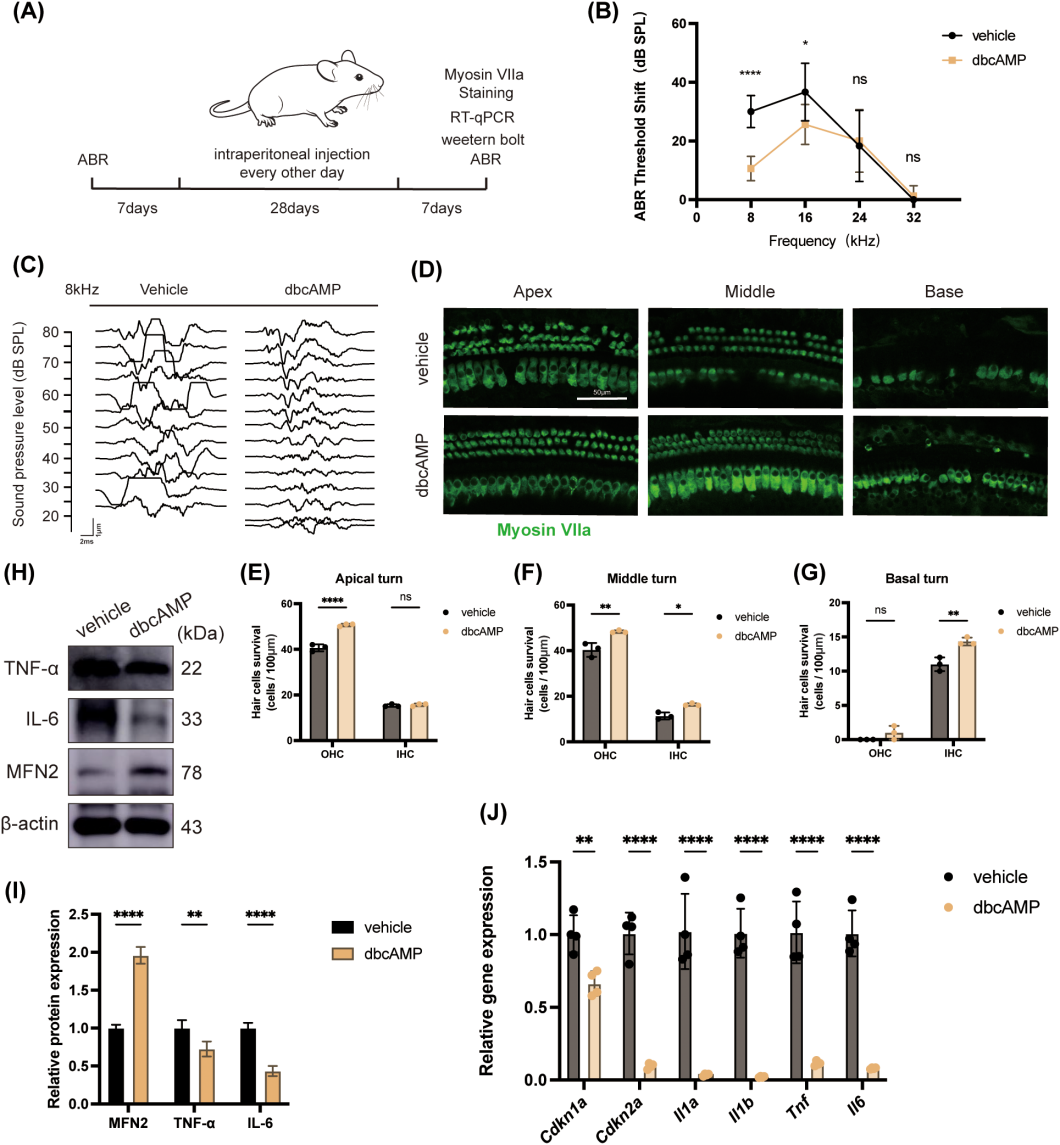
cAMP Treatment Rescues Auditory Function and Suppresses Cochlear Senescence in Aged Mice. Aged mice were initially divided into groups of ten. Due to mortality during the course of injections, the final analyses were performed on n = 6 mice in the vehicle group and n = 8 in the dbcAMP group. **(A)** Twenty male C57BL/6 mice (34–36 weeks old) were divided into saline (as a vehicle) and dbcAMP groups, with ABR measured before and after 28 days of intraperitoneal injection. **(B, C)** ABR results showing reduced threshold shifts in dbcAMP-treated mice at 8 and 16 kHz. n = 6 in the vehicle group and n = 8 in the dbcAMP group. **(D–G)** Myosin VIIa staining showing preserved OHCs and IHCs across apical, middle, and basal turns in saline-treated and dbcAMP-treated cochleae. Scale bar = 50μm; n = 3. **(H, I**) Western blot showing upregulation of MFN2 and reduced TNF-α/IL-6 expression following dbcAMP treatment. n = 3. **(J)** RT-qPCR results showing decreased expression of senescence- and inflammation-related genes (*Cdkn1a*, *Cdkn2a*, *Il1a*, *Il1b*, *Tnf*, *Il6*) in dbcAMP-treated mice. n = 4. Data are presented as mean ± SD. *p < 0.05, **p < 0.01, ***p < 0.001, ****p < 0.0001, n.s. no statistical difference; student’s t-tests.

We next investigated whether systemic administration of cAMP could mitigate age-related hearing loss. A cohort of twenty male C57BL/6J mice aged 34–36 weeks was initially included in the study and randomly divided into two treatment groups. During the 28-day treatment period of intraperitoneal injections with either saline or dbcAMP, several animals were excluded from the study due to health complications unrelated to the treatment. The final analysis of Auditory Brainstem Response (ABR) was therefore performed on 6 mice in the vehicle group and 8 mice in the dbcAMP group. Auditory function was assessed by ABR measurements before the treatment regimen and 7 days after its completion (**Figure 4A**).

Remarkably, dbcAMP treatment led to a significant preservation of hearing function. The auditory threshold shifts were markedly lower in the dbcAMP-treated group compared to the vehicle-treated group at both 8 kHz (10.63 ± 4.17 dB vs. 30 ± 5.48 dB, p < 0.0001) and 16 kHz (25.63 ± 6.78 dB vs. 36.67 ± 9.83 dB, p < 0.05) (**Figure 4B**). The post-treatment audiograms further illustrated that mice receiving dbcAMP exhibited significantly lower ABR thresholds at 8kHz compared to the vehicle group, indicating a substantial therapeutic effect (**Figure 4C**).

To determine the cellular basis for this functional improvement, we assessed the survival of cochlear hair cells, the primary mechanosensors for hearing. Immunofluorescence staining for MyosinVIIa, a specific marker for hair cells, revealed better preservation of both inner hair cells (IHCs) and outer hair cells (OHCs)in the apical, middle, and basal turns of the cochlea in the dbcAMP-treated mice, compared to the vehicle group (**Figures 4D–G**).

Finally, we sought to determine if the molecular mechanisms identified *in vitro* were recapitulated *in vivo*. Western blot analysis of cochlear protein showed that, consistent with our cell-based experiments, dbcAMP treatment led to a significant upregulation of MFN2 protein. Concurrently, the protein levels of pro-inflammatory cytokines TNF-α and IL-6 were markedly reduced in the cochleae of dbcAMP-treated mice (**Figures 4H, I**). These protein-level changes were further supported at the transcriptional level. qPCR analysis revealed that the expression of key senescence-related genes (*Cdkn1a*, *Cdkn2a*) and a broad panel of pro-inflammatory genes (*Il1a*, *Il1b*, *Tnf*, *Il6*) was significantly downregulated in the cochleae of the dbcAMP-treated group (**Figure 4J**).

In summary, these *in vivo* data robustly demonstrate that systemic cAMP administration can effectively ameliorate age-related hearing decline. This functional and structural protection is tightly associated with the upregulation of cochlear MFN2 and the suppression of senescence and inflammation, thus validating our *in vitro* findings in an animal model.

## 4 Discussion

In this study, we have identified a novel protective role for the cAMP signaling pathway in the context of age-related hearing loss. We provide compelling evidence that enhancing cAMP signaling potently suppresses cellular senescence and preserves auditory function in aged mice. Previous studies have shown that cAMP signaling declines with age and is associated with impaired PKA/CREB activation, mitochondrial dysfunction, and chronic inflammation (4–7). However, its role in the auditory system has remained largely unexplored. The central mechanism underlying this protection is the upregulation of Mitofusin 2 (MFN2), which we establish as an indispensable mediator of cAMP’s anti-senescence effects. Our findings delineate the cAMP-MFN2 axis as a critical regulatory node in auditory aging and propose it as a promising new therapeutic target for presbycusis.

A primary finding of our study is that exogenously increasing cAMP levels can potently counteract the hallmarks of cellular senescence and preserve auditory function in an *in vivo* model of aging. This finding aligns with a growing body of evidence that has established a crucial role for the cAMP pathway in regulating the aging process across a wide range of tissues (4–7). For instance, impaired cAMP signaling has been linked to the loss of cellular homeostasis and the development of human cataracts in the aging lens (4). In addition, reduced cAMP activity has been reported to exacerbate mitochondrial oxidative stress (17) and NF-κB–mediated inflammation (17, 18), both of which are strongly implicated in cochlear degeneration. Our work addresses this critical gap by demonstrating that enhancing cAMP signaling is a viable strategy to mitigate the cellular and functional deficits associated with presbycusis. Previous studies have shown that cAMP influences aging not only through classical PKA-CREB and exchange protein directly activated by cAMP (Epac) signaling, but also by modulating downstream effectors such as sirtuin 1 (SIRT1), AMP-activated protein kinase (AMPK), and forkhead box O (FOXO) transcription factors, which together regulate oxidative stress resistance, mitochondrial biogenesis, and cellular longevity (25–27). While cAMP is known for its wide-ranging cellular functions (19), our work underscores its powerful anti-senescence capacity within the vulnerable environment of the cochlea. These results are in line with previous observations in cardiovascular and neural aging models, where cAMP elevation improved mitochondrial homeostasis and suppressed inflammatory responses (7, 28). Consistent with these reports, our findings provide direct functional validation in the auditory system, confirming the crucial role of cAMP signaling in preserving mitochondrial bioenergetics under age-related stress. Together, these findings highlight the independent protective role of cAMP signaling in auditory aging, setting the stage for understanding its downstream mediators.

Our study also reveals that the protective effects of cAMP are closely associated with the regulation of MFN2. Our loss-of-function experiments unequivocally demonstrated that the beneficial effects of cAMP on cellular senescence were completely abrogated in the absence of MFN2. Although MFN2 is well-documented for its canonical role in mitochondrial fusion, a process critical for maintaining mitochondrial health and preventing cellular stress (28–30), our study provides new insights by linking its expression level to the regulation of senescence and inflammatory signaling in the auditory system. However, MFN2 is likely not the sole mediator of these effects. Other pathways may also be important contributors to the observed results. For example, elevated cAMP/PKA activity can stabilize OPA1 expression to preserve inner membrane fusion and cristae integrity, whereas PKA-dependent phosphorylation of dynamin-related protein 1 (DRP1) at Ser637 prevents its translocation to mitochondria and inhibits excessive fission (31, 32). These effects are further reinforced by Epac-mediated calcium signaling, which together ensure a proper balance between mitochondrial fusion and fission (16, 33). In our study, although we observed a significant increase in *Dnm1l* gene expression following dbcAMP treatment, the mitochondrial morphology showed a trend towards fusion. This phenomenon reveals the complexity of mitochondrial dynamics regulation. The *Dnm1l* gene encodes DRP1 protein, but the activity of DRP1 is primarily regulated by post-translational modifications, particularly phosphorylation (34). Therefore, despite the increase in *Dnm1l* gene expression, whether the activation state of DRP1 has changed requires further investigation. Additionally, the increase in *Dnm1l* gene expression may reflect a protective situation, where fission is necessary to remove damaged or dysfunctional mitochondria, which needs further study. Previous research has shown that MFN2 deficiency accelerates aging phenotypes, impairs oxidative phosphorylation, and promotes inflammatory signaling (35, 36), supporting the idea that MFN2 is a critical determinant of cellular fate during aging. This finding aligns with the emerging paradigm that positions mitochondria not just as metabolic hubs, but as central platforms for integrating signals that determine cell fate, including senescence and inflammation (37, 38). For instance, compromised mitochondrial integrity has been shown to trigger the cyclic GMP–AMP synthase–stimulator of interferon genes (cGAS–STING) pathway or activate the NOD-like receptor family pyrin domain-containing 3 (NLRP3) inflammasome, leading to a pro-inflammatory state (18, 39). By showing that cAMP enhances MFN2-dependent mitochondrial fusion, our study extends this paradigm by directly linking second messenger signaling to mitochondrial dynamics in the auditory system. Thus, our study forges a crucial link between cAMP signaling and MFN2-dependent control of cellular senescence, highlighting a key mechanism of auditory protection. Importantly, this control extends to suppressing pro-inflammatory signaling, representing a dual-pronged mechanism that is particularly relevant for presbycusis, a condition driven by the interplay of both cellular senescence and inflammaging (40, 41). Our *in vivo* data, showing a coordinated reduction of both senescence and inflammatory markers, strongly support this model.

Despite the robustness of our findings, this study has several limitations that warrant consideration. First, our study has limitations related to the *in vitro* models used. Our experiments were conducted in the HEI-OC1 cell line, which, while a valuable tool, may not fully replicate the physiological characteristics of primary cochlear cells, such as their cellular heterogeneity, differentiation state, and responsiveness to the cochlear microenvironment (42). Furthermore, we employed a D-galactose-induced senescence model. This model effectively mimics certain aspects of aging, such as oxidative stress and inflammation (43, 44), but its acute nature does not capture progressive aging processes, including telomere attrition, mitochondrial DNA mutations, or long-term cellular remodeling (45). The validation of our findings in more physiologically relevant models, therefore, remains an important avenue for future investigation. Second, a key unanswered question from our study is the precise molecular mechanism by which cAMP signaling upregulates MFN2 expression. Our work establishes a clear functional link, but the intermediary signaling components that connect cAMP to the transcriptional regulation of *Mfn2* remain to be identified. Previous studies have shown that activation of CREB can upregulate MFN2 expression and enhance mitochondrial homeostasis (20). Considering that cAMP signaling activates CREB through the classical PKA pathway, it is plausible that dbcAMP increases MFN2 expression in cochlear cells via CREB regulation. This regulatory axis may provide a mechanistic link between cAMP signaling and mitochondrial fusion, thereby contributing to the anti-senescence and auditory protective effects observed in our model. Elucidating this pathway will be an important focus of future research. Third, we acknowledge a limitation regarding the interpretation of our *in vivo* findings due to the systemic administration of dbcAMP. While our *in vitro* data provide strong evidence for a direct protective mechanism within cochlear cells, it is challenging in the context of a whole animal model to definitively exclude the possibility that indirect systemic effects, such as improvements in the overall inflammatory tone or metabolic status, may have contributed to the preservation of hearing. Future studies employing local administration methods, such as round window membrane application, or the use of conditional genetic models to modulate the cAMP-MFN2 axis specifically in hair cells, will be necessary to dissect the direct versus indirect contributions *in vivo*. Fourth, a limitation of this study is that we used the C57BL/6J strain, which carries the *Cdh23* mutation and is known for exhibiting early, high-frequency hearing loss. While this strain is commonly used as a model for age-related hearing loss, future studies should validate the findings in other strains, such as CBA/CaJ, to strengthen the generalizability of our conclusions.

In conclusion, our study elucidates a previously unrecognized cAMP-MFN2 axis that functions as a critical defense mechanism against cellular senescence in the aging auditory system. By demonstrating that enhancing cAMP signaling preserves hearing function in aged mice, we have provided a strong preclinical rationale for therapeutically targeting this pathway. Future directions should focus on deciphering the precise molecular cascade linking cAMP to MFN2 and on developing targeted strategies to modulate this pathway within the inner ear. Ultimately, our findings identify a novel potential target for interventions aimed at slowing the progression of age-related hearing loss. This condition profoundly impacts the quality of life for millions worldwide.

## Data Availability

The original contributions presented in the study are included in the article/**Supplementary Material**. Further inquiries can be directed to the corresponding authors.
