# Supplementary material for: cAMP-MFN2 signaling suppresses cochlear cell senescence and age-related hearing loss
Source: Front Immunol. 2025 Nov 26;16:1715738. doi: 10.3389/fimmu.2025.1715738 (PMC12689285; doi:10.3389/fimmu.2025.1715738)
Supplement: Supplementary file 2 [file Table2.docx]

Raw Data:

https://www.jianguoyun.com/p/DVoDTiUQ6vTeDRjd34kGIAA
