# Supplementary material for: cAMP-MFN2 signaling suppresses cochlear cell senescence and age-related hearing loss
Source: Front Immunol. 2025 Nov 26;16:1715738. doi: 10.3389/fimmu.2025.1715738 (PMC12689285; doi:10.3389/fimmu.2025.1715738)
Supplement: Supplementary file 3 [file Table3.docx]

Checklist

1. manuscript
2. figure1, 2, 3, 4
3. table s1
4. Raw Data
5. Ethics Approval
